# Evaluating morphometric and metabolic markers of body condition in a small cetacean, the harbor porpoise (*Phocoena phocoena*)

**DOI:** 10.1002/ece3.2891

**Published:** 2017-04-09

**Authors:** Joanna L. Kershaw, Meredith Sherrill, Nicholas J. Davison, Andrew Brownlow, Ailsa J. Hall

**Affiliations:** ^1^ Sea Mammal Research UnitScottish Oceans InstituteUniversity of St AndrewsSt AndrewsFifeUK; ^2^ Scottish Marine Animal Stranding SchemeSAC Veterinary Services DrummondhillInvernessUK

**Keywords:** blubber biopsies, body condition index, cetaceans, cortisol, mass/length^2^

## Abstract

Mammalian body condition is an important individual fitness metric as it affects both survival and reproductive success. The ability to accurately measure condition has key implications for predicting individual and population health, and therefore monitoring the population‐level effects of changing environments. No consensus currently exists on the best measure to quantitatively estimate body condition in many species, including cetaceans. Here, two measures of body condition were investigated in the harbor porpoise (*Phocoena phocoena*). First, the most informative morphometric body condition index was identified. The mass/length^2^ ratio was the most appropriate morphometric index of 10 indices tested, explaining 50% of the variation in condition in stranded, male porpoises with different causes of death and across age classes (*n* = 291). Mass/length^2^ was then used to evaluate a second measure, blubber cortisol concentration, as a metabolic condition marker. Cortisol is the main glucocorticoid hormone involved in the regulation of lipolysis and overall energy balance in mammals, and concentrations could provide information on physiological state. Blubber cortisol concentrations did not significantly vary around the girth (*n* = 20), but there was significant vertical stratification through the blubber depth with highest concentrations in the innermost layer. Concentrations in the dorsal, outermost layer were representative of concentrations through the full blubber depth, showed variation by sex and age class, and were negatively correlated with mass/length^2^. Using this species as a model for live cetaceans from which standard morphometric measurements cannot be taken, but from which blubber biopsy samples are routinely collected, cortisol concentrations in the dorsal, outermost blubber layer could potentially be used as a biomarker of condition in free‐ranging animals.

## 1 Introduction

Mammalian body condition is commonly defined as the quantity of energy carried in an individual's lipid stores (Pitt, Larivière, & Messier, 2006). The size of these stores reflects the integration between foraging effort, foraging success, and reproductive needs throughout the life cycle (Aguilar & Borrell, 1990). In marine mammal ecology, body condition has been demonstrated to be a good predictor of fitness as it affects both survival and reproductive success (Pettis et al., 2004; Pitcher, Calkins, & Pendleton, 1998). Thus, understanding variation in body condition among individuals within a population and how this can influence demographic processes is important for predicting population health and resilience to external stressors.

In terrestrial studies investigating condition, ecologists have developed a number of nondestructive methods using the relationships between body mass and morphometric measurements to interpret variations in mass for a given size (Peig & Green, 2010). A wide range of different formulas and statistical methods have been proposed to standardize measurements, but there is still much debate about which are the most suitable (Peig & Green, 2010). In cetaceans, lipid energy reserves are stored in the blubber, and for this reason, it has previously been assumed that blubber thickness is a good indicator of condition (Koopman et al., 2002; Lockyer, McConnell, & Waters, 1985). Other common indices used in cetaceans are maximum girth, length, and mass (Read, 1990; Víkingsson, 1995). More recent efforts have considered different scaling relationships between these measures (Hart, Wells, & Schwacke, 2013), and estimations of total blubber volume (Christiansen, Víkingsson, Rasmussen, & Lusseau, 2013). However, despite its importance, little is known about the body condition of many cetacean species, and currently, no consensus has been reached about the best morphometric condition index, or criteria which allow the objective determination of the most appropriate calculation of an index for any given species or a particular study.

In addition, robust, practical alternatives for when morphometric measurements cannot be obtained, as is the case for the majority of live cetacean species, also require investigation. For example, while smaller cetaceans can be temporarily restrained for the collection of morphometric measurements (Adams et al., 2014; Wells et al., 2004), there are few methods for assessing body condition in larger species that cannot be handled. As a potential alternative, physiological markers could be a valuable, objective, and widely applicable tool for estimating the body condition of cetaceans provided appropriate biomarkers can be identified. One such alternative is the use of endocrine markers.

Cortisol is a lipophilic steroid that has the potential to be an informative marker of nutritive condition as it is the primary glucocorticoid hormone involved both in the stress response and, importantly in this context, in the regulation of mammalian energy balance (Sapolsky, Romero, & Munck, 2000; Strack, Sebastien, Schwartz, & Dallman, 1995). To date, investigation of this hormone in marine mammal energy regulation and fasting metabolism has focused on pinnipeds (Guinet, Servera, Mangin, Georges, & Lacroix, 2004; Kershaw & Hall, 2016; Ortiz, Houser, Wade, & Ortiz, 2003). In cetaceans, three studies have measured cortisol concentrations in blubber with emphasis on its variation with blubber depth (Trana, Roth, Tomy, Anderson, & Ferguson, 2015), and its use to investigate the stress response (Kellar et al., 2015; Trana, Roth, Tomy, Anderson, & Ferguson, 2016). However, cortisol also plays an active role in lipolysis in subcutaneous adipose tissue (Divertie, Jensen, & Miles, 1991; Djurhuus et al., 2004; Samra et al., 1998), so cortisol concentrations in the blubber could also provide information about physiological state in terms of lipid mobilization and deposition. This is of particular relevance to the analysis of blubber biopsy samples from free‐ranging individuals which has become a widespread method for obtaining cetacean tissue samples (Hunt et al., 2013).

The aim of this study was therefore to identify the most appropriate morphometric condition index in a small odontocete species, the harbor porpoise (*Phocoena phocoena*), and to compare this index to blubber cortisol concentrations as an alternative physiological, endocrine marker of condition. Here, morphometric data collected from 291 stranded, male harbor porpoise by the Scottish Marine Animal Strandings Scheme were analyzed to identify a morphometric index most diagnostic of nutritional status. Then, full‐depth blubber samples collected from 20 individuals were analyzed to investigate the potential use of blubber cortisol concentrations as an informative condition biomarker.

## 2 Materials and Methods

### 2.1 Morphometrics dataset

#### 2.1.1 Data collection

Data collected by the Scottish Marine Animal Strandings Scheme (SMASS) from stranded male harbor porpoises (*n* = 291) around Scotland, UK, were collated between January 2006 and January 2016 to assess different morphometric measures of body condition. For these animals, the cause of death (COD) was determined either by necropsy, or based on visual observations of the carcass showing signs of trauma, disease, or emaciation. Measurements of mass, length, girth, and blubber thickness proximal to the dorsal fin along the dorsal, lateral, and ventral axes were also taken (Kuiken & Hartmann, 1991). Females were not included in the analysis as the reproductive status (pregnant, lactating, or resting) was not known for all individuals sampled over the same 10‐year period. Reproductive status would likely have affected the estimated condition of the individuals as pregnant females increase in both mass and girth irrespective of their condition. Thus, without knowing the pregnancy status of females, all metrics that contain mass and girth are inherently confounded as they represent changes in both body condition and pregnancy status.

The 291 individual males were grouped into two COD categories. “Acute” cases (*n* = 133) were individuals that died of an acute trauma (bycatch, entanglement, storm damage, and bottlenose dolphin attacks), and “chronic” cases (*n* = 158) were individuals that died of general debilitation either through infectious disease (parasitic, bacterial, viral, or mycotic infections) or starvation (severely emaciated animals that died of starvation/hypothermia). The individuals were grouped into age classes based on the size of males at sexual maturity. Adults were ≥135 cm (Ólafsdóttir, Víkingsson, Halldórsson, & Sigurjónsson, 2002), calves were ≤90 cm (Lockyer, 1995a), and others were classed as juveniles.

These data were further grouped into two life‐history stages. As the peak calving months are June and July in the North Sea, and a spike in male testes mass in August suggests that mating occurs largely in this month (Lockyer, 1995a), data from June, July, and August were grouped as “breeding” (*n* = 66) and the rest of the year was considered “nonbreeding” (*n* = 225).

#### 2.1.2 Statistical analysis

All statistical analyses were performed using the statistical package, R, version 3.1.2 (R Core Development Team, 2014).

The most representative morphometric index of body condition was investigated based on three ground‐truthing assumptions:

1. “Chronic” cases will have a lower body condition index than “acute” cases because the acute cases died as a result of critical physical injury and did not become debilitated with a gradual decline in their health.
2. Overall, juveniles will have a lower condition index than adults. These individuals have recently weaned and are foraging independently for the first time making them vulnerable to nutritional stress. This age group is also most likely to be physiologically stressed due to immunological challenge by parasites encountered for the first time, and any extra energy acquired is likely used for growth rather than retained as fat stores.
3. Body condition in the breeding season will be lower than the rest of the year as males expend energy on reproduction.

Thus, a reliable condition index should be able to differentiate between individuals with different CODs, age classes, and life‐history stages. Seven body condition indices commonly used to assess condition in mammals were calculated based on previous work by Peig and Green (2009, 2010; Table 1). Ventral blubber thickness was also investigated as a potential index as it has been previously identified as a dynamic lipid storage area in this species (Koopman, Pabst, McLellan, Dillaman, & Read, 2002). Two additional condition metrics, ventral blubber thickness/girth and ventral blubber thickness/length, were also assessed as potentially more useful indices than blubber thickness alone that will vary as a function of overall size of the individual irrespective of its relative condition.

**Table 1:** Body condition indices and their calculation. With the exception of ventral blubber thickness, ventral blubber:girth, and ventral blubber:length, formulae are taken from Peig and Green (2010)

Index	Formula and explanation
Ventral blubber thickness	Full blubber depth
Ventral blubber:girth	Full ventral blubber depth/girth
Ventral blubber:length	Full ventral blubber depth/length
Girth:length	Girth/length
Mass:length	Mass/length
Fulton's index	*K* = mass/length^3^
Quetelet's index	BMI = mass/length^2^
Relative condition	$K_{n}\;=\;M_{i}/M_{i}^{*}$ (individual mass/predicted mass)
	$M_{i}^{*}\;=\;aL_{i}^{b}$ where *a* and *b* are determined by ordinary least squares (OLS) regression of *M* against *L* for the whole study population
Residual index	*R*_i_, the residuals from an OLS regression of *M* against *L*, after log transformation
Scaled mass index	${\hat{M}}_{i}\;=\;M_{i}\;{\left[\frac{L_{o}}{L_{i}}\right]}^{b_{\mathrm{SMA}}}$
	Where *M*_i_ and *L*_i_ are the mass and length of an individual. ${{}^{b}}_{\mathrm{SMA}}$ is the scaling component estimated by the standardized major axis (SMA) regression of ln *M* on ln *L*
	*L*_0_ the arithmetic mean of the length of the study population
	${\hat{M}}_{i}$ is the predicted body mass of the individual when the linear body measure is standardized to *L*_0_

The condition of an individual was assumed to be independent of the condition of others, there was no evidence of different standard deviations across the COD classes, age classes, or season groups for each index (leveneTest in the *Rcmdr* R package to test for homogeneity of variance between groups, *p* values all >.05), and all indices were normally distributed. Thus, linear regression models were used to investigate the relationship between the index and the suite of covariates discussed above as well as an interaction between season and age class. Backwards model selection was performed using the *dredge* function (*MuMIn* package in R) which identifies the variables and/or interactions that best explain the variation in the data. *Dredge* performs automated model selection with subsets of a supplied “global” model including all covariates and interactions of interest, and optional choices of other model properties (such as different link functions). All possible combinations of covariates and their interactions were generated, and the model with the lowest Akaike information criterion (AIC) value with at least a value of two smaller than the next smallest AIC value was identified as the best model for each index and was used for further interpretation. For each index, the model with the smallest AIC met these criteria, so there were no models that would have been considered of equivalent fit to the data.

Visual inspections of the model residual plots and fitted values were used to check the fit and assumptions of the final models. When residuals are plotted against the fitted values, if the model is suitable and has not violated any major assumptions, there should be no obvious patterns or trends across the data points. If the model fits well, the fitted values should be similar to the observed values and when plotted together would lie approximately along the 45° line. If this is not the case, there may be some autocorrelation in the data and some over‐ or underprediction occurring in the model.

The relationships between the covariates retained after model selection for each index were evaluated, and the model fit was assessed using the adjusted *R* ^2^. As well as evaluating the model fit, the variance explained by each variable retained in the final models was also assessed. When there is more than one predictor, the partial eta‐squared (partial η^2^) is the proportion of the variance explained by a given predictor after excluding variance explained by the others (Levine & Hullett, 2002). For each body condition index model, we used partial η^2^ to assess the importance of each variable retained after model selection. Partial η^2^ values were calculated using the “*etasq*” function in the *heplots* library in R; the sums of squares for the effect of interest were divided by the combined sums of squares of the effect of interest and the associated error term.

The condition index that showed the expected relationships based on the ground‐truthing assumptions and was a good fit to the data (a high adjusted *R* ^2^) and thus good predictive power, was then used in the following analyses to investigate differences in cortisol concentrations in the blubber samples.

### 2.2 Blubber cortisol concentrations

#### 2.2.1 Sample collection

Full‐depth skin, blubber, and underlying muscle samples were collected from 20 dead harbor porpoise by the SMASS between 2013 and 2015. Only freshly dead animals, classified as those that originally stranded alive or had only recently died and thus showed no evidence of bloating, and the meat is considered to be edible (Kuiken & Hartmann, 1991), were sampled for this work in order to prevent erroneous hormone concentration measurements as a result of tissue decomposition after death. Samples were collected from the dorsal, lateral, and ventral axes around the girth of the animal, immediately caudal to the dorsal fin. Blubber depth was recorded at each sampling site. Tissue samples were individually wrapped in aluminum foil and stored at −20°C in plastic vials before hormone analysis.

These individuals were both adults (*n* = 13) and juveniles (*n* = 7), and males (*n* = 11) and females (*n* = 9). The COD was determined following postmortem examination and classified, as for the morphometric dataset, as either an acute (*n* = 13) or chronic (*n* = 7) COD.

#### 2.2.2 Blubber sample processing and cortisol extraction

Subsamples (0.1–0.2 g) were taken from each sample while still frozen and used for cortisol extraction. Full‐depth blubber subsamples were taken from the three sampling sites (dorsal, lateral, and ventral) for all 20 animals. Subsamples of the outer, middle, and inner blubber layers were taken across these sampling sites for six of the 20 individuals. For thicker blubber samples (depth ≥ 15 mm), all subsamples were 5 mm in depth with the outer‐layer subsamples taken immediately below the skin and inner‐layer subsamples taken immediately above the muscle layer. The middle‐layer subsamples were taken immediately above the inner‐layer subsamples. For thinner samples (≤15 mm depth), the blubber thickness was divided into three equal parts to give outer middle and inner layer subsamples of equal thickness. Finally, the outer layer only was subsampled from the dorsal site of all 20 individuals. The steroid extraction method described by Kellar, Trego, Marks, and Dizon (2006) was used to extract cortisol from the subsamples.

#### 2.2.3 Cortisol quantification

Prior to analysis, the final extraction residues were centrifuged briefly and then resuspended in 500 μl of phosphate‐buffered saline (pH 7.5). Cortisol concentrations were determined using a commercially available enzyme‐linked immunosorbent assay (ELISA) kit (DRG International Inc., Marburg, Germany: Cortisol ELISA EIA‐1887). This kit has been used to quantify cortisol in blubber biopsies from harbor seals (*Phoca vitulina*; Kershaw & Hall, 2016). Cortisol concentrations were measured according to the ELISA kit instructions with a standard curve ranging from 0 to 800 ng/ml, with a sensitivity of 2.5 ng/ml. The hormone concentrations in the samples were calculated from the standard curve using a four‐parameter log‐logistic model. All extracts were above the limit of detection and were assayed in duplicate, and the mean hormone concentration was reported in ng/g.

Extracts of varying concentrations (*n* = 10) were used to calculate interassay and intraassay coefficients of variation (CV), with mean percentage CVs of <20% and <10% set as the acceptable limits, respectively (Andreasson et al., 2015). The mean interassay CV was 10.84%, and the mean intraassay CV was 5.65%.

#### 2.2.4 Assay and extraction verifications

Parallelism assays were used to validate the use of this ELISA with harbor porpoise blubber extracts (Appendix S1). PBS was confirmed to be a compatible sample diluent with this cortisol ELISA kit in previous work with blubber extracts (Kershaw & Hall, 2016). Limitations and sources of error in the cortisol extraction method were also assessed to correctly interpret the results and assess the potential use of this method for remotely obtained biopsy samples. Firstly, the extraction efficiency was assessed across tissue samples of different masses using spiked recovery tests (Appendix S1). Secondly, the minimum sample mass required to obtain robust, replicable measurements of cortisol concentrations in the blubber samples was assessed through multiple extractions from the same piece of tissue of differing masses (Appendix S1).

#### 2.2.5 Statistical analysis

Two different modeling approaches were used to assess, first the effects of both sampling site and sampling depth on blubber cortisol concentration and, second, the effects of other explanatory covariates (age class, sex, COD, and body condition). A summary of each set of subsamples used for the various models is given in Table 2.

**Table 2:** Summary of each sample subset used for three different models

Number of individuals	Body location	Blubber layer	Model
20	Dorsal, lateral, ventral	Full	GLMM of body location
6	Dorsal, lateral, ventral	Full, outer, middle, inner	GLMM of body location and layer together
20	Dorsal	Full, outer	GLM of cortisol covariates

GLMM, generalized linear mixed‐effect model; GLM, generalized linear model.

##### Body location and blubber layer

Generalized linear mixed‐effect models (GLMMs) were used to investigate cortisol concentrations both across body locations and through the blubber depth while accounting for the repeated measurements from the same individuals that are considered as random effects (Bolker et al., 2008). Two GLMMs (*glmer* function in the R package *lme4*) with a gamma distribution to better model the right skew in the cortisol concentration data, a log link function, and each individual treated as a random effect were used to investigate, first, the effect of body location on cortisol concentration in full‐depth subsamples (*n* = 20 individuals with three samples each, Table 2) and, second, the effect of body location *and* blubber layer (*n* = 6 individuals consisting of 3 females and 3 males, 4 acute cases and 2 chronic cases with 12 samples each, Table 2). An interaction between body condition (mass/length^2^) and location, as well as between body condition and layer, was also included to consider the possibility that animals in varying condition may show differences in their cortisol distribution through the blubber depth and across their bodies as a result of differential energy store mobilization. Backwards model selection using the *dredge* function was used again to identify the variables and/or interactions that best explain the variation in hormone concentrations and thus to include in the final models. The goodness of fit of each model was assessed using the AICc (second‐order AIC which uses a correction for finite sample sizes). The model with the lowest AICc value was used for further interpretation.

##### Cortisol covariate analysis

Generalized linear models (GLMs) were used to assess the effects of other variables on the cortisol concentrations measured in the outer layer of the dorsal samples as those that are representative of biopsies taken from live animals (*n* = 20; Table 2). GLMs were used to better model the right skew in hormone concentrations using a gamma distribution and a log link function. The largest GLM including all the explanatory variables (age class, sex, COD category, and body condition) was generated. Again, backwards model selection using the *dredge* function was used to identify the variables that best explain the variation in hormone concentrations, and thus to include in the final model based on the smallest AICc. As described for the linear models used for the morphometric dataset, visual inspections of the model residual plots and fitted values from the final model gave confidence in the results and allowed interpretation of the model coefficients to assess the effects of each covariate.

## 3 Results

### 3.1 Morphometric data analysis

The interaction between age class and season was not retained in any of the linear models. The predominant COD varied by age class. Fewer calves died as a result of trauma (25%) compared to both juveniles and adults (47.8% and 49.1%, respectively). There was a significant effect of COD on all body condition indices (Table 3). All indices showed that the chronic cases were in poorer condition than the acute cases, as hypothesized based on the ground‐truthing assumptions, but the results varied by age class. Five of the indices suggested that adults were in better condition than the other age classes, and the other five suggested that there was either no effect or they were in poorer condition (Table 3).

**Table 3:** Summary of the covariates used in the linear regression models for each body condition index

Body condition index model		Model covariates used in model selection			Model adjusted *R* ^2^
		COD	Age class	Season	
Ventral blubber thickness	Variable retained	a	a	a	0.22
	Assumptions met	+	+	+	
	Partial η^2^	0.15	0.06	0.01	
Ventral blubber/girth	Variable retained	a	a	a	0.09
	Assumptions met	+	−	+	
	Partial η^2^	0.14	0.05	0.01	
Ventral blubber/length	Variable retained	a	a	a	0.15
	Assumptions met	+	−	+	
	Partial η^2^	0.14	0.05	0.01	
Girth/length	Variable retained	a		a	0.19
	Assumptions met	+		−	
	Partial η^2^	0.19		0.01	
Mass/length	Variable retained	a	a		0.72
	Assumptions met	+	+		
	Partial η^2^	0.17	0.69		
Fulton's index	Variable retained	a	a		0.17
	Assumptions met	+	−		
	Partial η^2^	0.13	0.08		
Quetelet's index	Variable retained	a	a		0.50
	Assumptions met	+	+		
	Partial η^2^	0.19	0.40		
Relative condition	Variable retained	a	a		0.25
	Assumptions met	+	+		
	Partial η^2^	0.17	0.08		
Residual index	Variable retained	a	a		0.21
	Assumptions met	+	+		
	Partial η^2^	0.11	0.17		
Scaled mass index	Variable retained	a			0.14
	Assumptions met	+			
	Partial η^2^	0.14			

If ground‐truthing assumptions were met, the relationships are marked with a “+,” while different relationships are marked with “−.” Partial η^2^ is the proportion of variance explained by that variable in the final model after excluding variance explained by the other variables. COD, cause of death.

The variable was retained after model selection.

a The variable was retained and statistically significant (*p* < .05).

As it is unlikely that overall, adults would have smaller energy reserves than both juveniles and calves, these condition indices (blubber thickness/girth, blubber thickness/length, and Fulton's index) were not used for further interpretation. As girth/length and the scaled mass index showed no differences between age classes, these were also dropped for further interpretation. Of the remaining indices (blubber thickness, mass/length, Quetelet's index, relative condition, and the residual index), there was little variation in the effect size for COD between the final models for each index with partial η^2^ values ranging from 0.11 to 0.19 (Table 3). However, there was more variation in the effect size for age class between these models, with partial η^2^ values ranging from 0.06 to 0.69 (Table 3). The model for mass/length was the best fit to the data with an adjusted *R* ^2^ value of 0.72 (Table 3), but, as seen with the partial η^2^ values for COD and age class, the variation is largely explained by the age class, and thus the different size of individuals.

**Figure 1:**
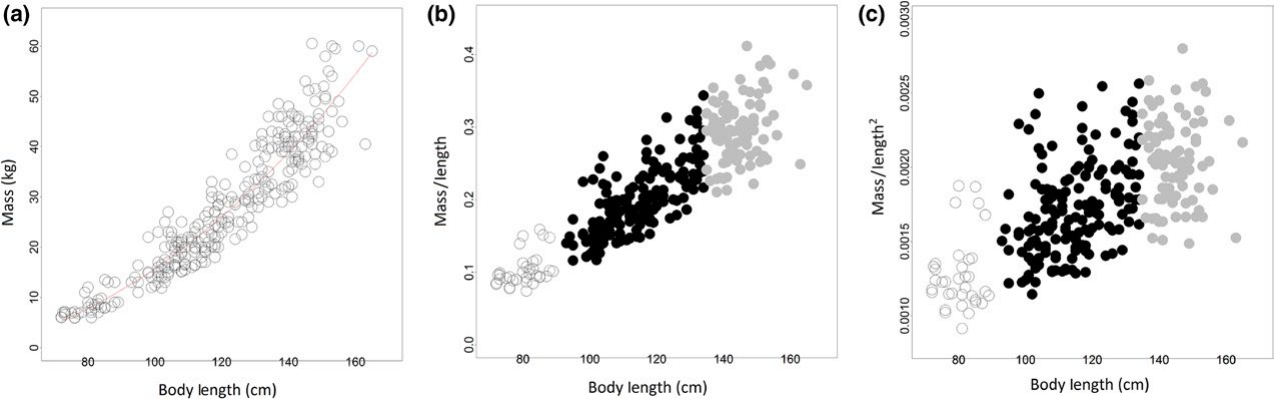
Relationships between mass and length and their ratios for male harbor porpoises. (a) Nonlinear scaling relationship between mass and length for male harbor porpoises modeled using nonlinear least squares regression where mass = −3.71 × 0.0004 length^2.33^. (b) Positive correlation between mass/length and length demonstrating that using mass/length as a condition index is not independent of body size. (c) Relationship between mass/length^2^ and length demonstrating that mass/length^2^ is a more appropriate index for this species to compare between adults and juveniles as it is largely independent of body size

Generally mass shows a strong nonlinear relationship with length, which was also the case for these male porpoises (Figure 1a), such that much of the variation in mass is accounted for by changes in length (Hayes & Shonkwiler, 2001). Thus, indices of condition that are not explicitly based on allometric models are affected implicitly by the scaling of mass and length (Hayes & Shonkwiler, 2001). For this reason, the simplest mass/length condition index, identified here as the best fit to the data, is correlated with size (Figure 1b). Mass/length as a condition index is therefore flawed because it inherently incorporates an association between length and condition and can therefore be misleading because the effects of condition are likely confounded with the effects of size for this species.

The second best‐fitting model for these data was the Quetelet's index (mass/length^2^) with an adjusted *R* ^2^ value of 0.50. In addition, for mass/length^2^, the effect size for COD was highest with a partial η^2^ value for COD of 0.19. Thus, using differences in COD as the most relevant ground‐truthing assumption for detecting changes in condition, this index explains the most variation in the COD data. In general, the relationship between mass and length can be modeled as (Hayes & Shonkwiler, 2001):

$$\text{mass}\;=\;\delta \;+\;\alpha \;{\text{length}}^{\beta }.$$

This equation shows that mass is a power function of length plus an intercept term, and the parameters of this equation can be estimated empirically by nonlinear regression. When the goal is to generate a condition index that is independent of size, use of the mass/length ratio may be reasonable if mass scales with length such that β = 1 and δ is close to 0 (Hayes & Shonkwiler, 2001). If the scaling exponent does not equal 1, then obtaining a size‐independent condition ratio requires that some scaling exponent other than 1 should be used.

**Figure 2:**
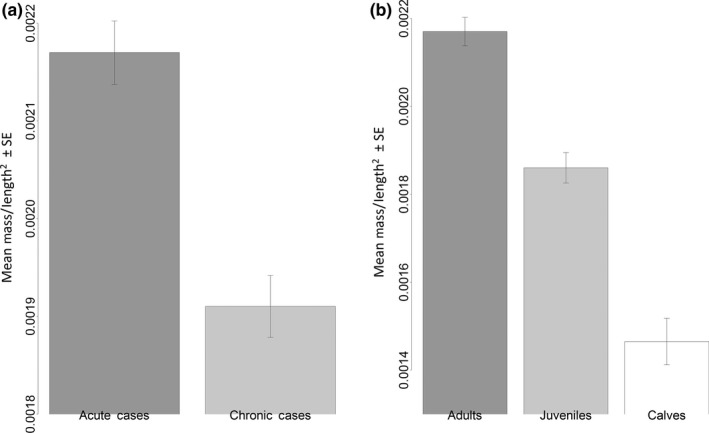
Output from the final mass/length^2^ linear regression model: lm(Mass.Length^2^ ~ as.factor(COD)+as.factor(Age.Class)) (*n* = 291). (a) The chronic cases had a significantly lower mass/length^2^ index than the acute cases (*p* value < .005). (b) Adults had the highest mass/length^2^ index of all three age classes (*p* value < .005)

The above nonlinear model was fit to the harbor porpoise data (*nls* function in the R package *nlstools*; Figure 1a). The δ parameter estimate was −3.17 with large 95% CIs of −9.10 to 1.66. As a negative intercept is not biologically plausible, this is likely driven by a lack of data for very small calves and fetuses. As this nonlinear model would not be used to extrapolate beyond the smallest individuals measured here, and the intercept parameter estimate confidence interval incorporates 0, the intercept is assumed to be around 0. The β exponent was estimated as 2.33 (with 95% confidence intervals of 1.89–2.77), which suggests that while mass/length^2^ showed a poorer overall fit to the data, it is a better index to use here in order to obtain an index largely independent of body size (Figure 1c). Using this index, the condition of adults and juveniles can be better compared (Figure 1c), but the condition index of calves compared to the other two age classes should be interpreted with caution. Using mass/length^2^, acute cases were in significantly better condition than the chronic cases (*p* value < .005) (Figure 2a), and adults were in the best condition and calves in the poorest (*p* values all < .005; Figure 2b), but season was not retained in the best‐fitting model (Table 3).

### 3.2 Cortisol data analysis

#### 3.2.1 Parallelism assessment

Parallelism between blubber extract dilutions and the standard curve confirmed that this ELISA is suitable for the quantification of harbor porpoise cortisol (Appendix S1).

#### 3.2.2 Extraction efficiency and minimum sample mass

The calculated extraction efficiencies were used to correct the measured cortisol concentrations in each sample to give a final cortisol concentration used for statistical analysis (Appendix S1). Both extraction efficiency and measurement variability decreased with increased sample mass. Based on these relationships, it is recommended that for maximum extraction efficiency and minimum variability, blubber samples should be between 0.15 and 0.2 g (Appendix S1).

#### 3.2.3 Body location and blubber layer

**Figure 3:**
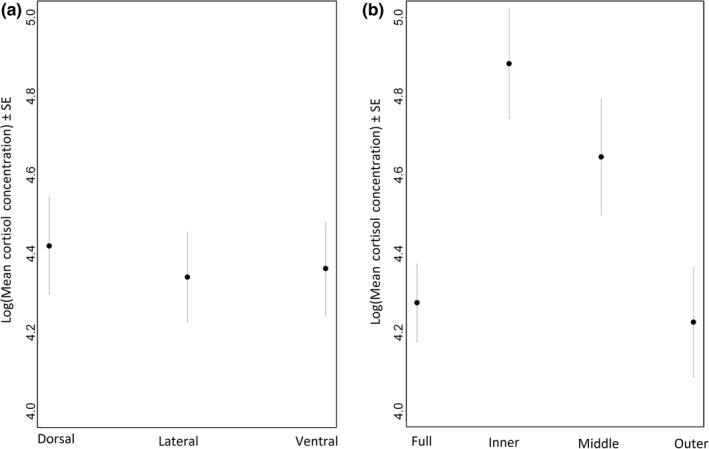
Final generalized linear mixed‐effect model (GLMM) outputs for cortisol concentrations across the body and through the blubber layer. (a) GLMM output for blubber cortisol concentrations around the girth. There were no significant differences in cortisol concentration in full‐depth blubber samples from three different sampling locations. (b) GLMM output following variable selection for blubber cortisol concentrations with both location and blubber depth. The inner and middle layers had significantly higher cortisol concentrations than the full depth and outer layers (*p* values < .01), while the outer layer was not significantly different to the full depth sample overall

Blubber cortisol concentrations ranged from 3.65 to 759.51 ng/g. The final GLMMs following variable selection did not retain either body location or the interaction with body location and body condition as significant explanatory variables. Mean concentrations of 69.09 ± 31.30, 90.48 ± 52.22, and 83.22 ± 59.95 ng/g were measured in the full‐depth dorsal, lateral, and ventral samples, respectively. Thus, there are no significant differences in blubber cortisol concentrations across these three different sampling locations (Figure 3a). When blubber layer was considered together with body location and condition, the final GLMM following variable selection retained only blubber layer as an important explanatory variable. This suggests that the pattern of cortisol distribution through blubber depth does not change with sampling site, or animal condition. The highest concentrations were measured in the inner and middle layers (means of 180.02 ± 177.06 and 156.28 ± 167.02 ng/g, respectively; *p* values < .01; Figure 3b), and there was no significant difference between the concentrations in the full‐depth samples compared to the outer layer alone (means of 88.65 ± 68.38 and 77.84 ± 48.32 ng/g, respectively; Figure 3b).

#### 3.2.4 Cortisol covariates

**Figure 4:**
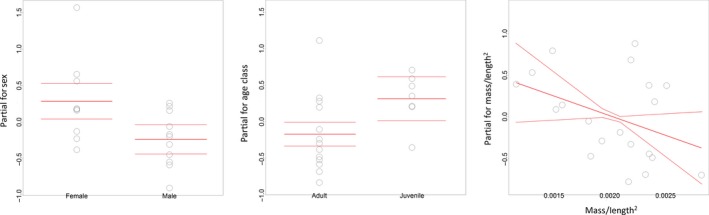
Partial termplots for the covariates retained in the final two best‐fitting generalized linear models following variable selection: glm(Outer.Cort ~ Sex + Age.Class, family = Gamma(link = “log”)) and glm(Outer.Cort ~ Sex + Mass/Length^2^, family = Gamma(link = “log”)) (*n* = 20). Termplots plot regression terms against their predictors with the associated standard errors, while holding other predictors at their mean values. Significantly higher cortisol concentrations were measured in females than in males (*p* = .02), and in juveniles than in adults (*p* = .05). There was a weakly significant negative relationship between mass/length^2^ and blubber cortisol concentration (*p* = .1)

Variable selection for the GLM of cortisol concentration in the dorsal, outer‐layer samples revealed that there was less than a two‐point difference in the AICc between the two best‐fitting models, indicating that they were of equivalent fit to the data (Table 4). These models retained both age class and sex, and body condition and sex as important explanatory variables (Table 4). Juveniles had higher cortisol concentrations than adults (*p* = .05), and there was a weakly significant negative relationship between mass/length^2^ and cortisol concentration (*p* = .1). Overall, females had significantly higher cortisol concentrations than males with means of 111.37 ± 59.86 ng/g compared to 66.77 ± 29.73 ng/g (*p* = .02; Figure 4). This could be as a result of the cross‐reactivity of the ELISA kit with progesterone (19%). However, one adult female that died as a result of dystocia (classed here as an acute case) did not have an elevated blubber cortisol concentration (53.90 ng/g) as would be expected if there were high levels of blubber progesterone associated with pregnancy (Trego et al., 2013). Nonetheless, to investigate the potential confounding effect of this cross‐reactivity, the male and female data were modeled separately with the same covariates and model selection process. For the male dataset, again, the final model selection showed that there was less than a two‐point difference in the AICc between the two best‐fitting models including both age class and mass/length^2^ although neither was individually significant. For the female dataset, variable selection excluded all covariates, and these results are likely a result of the small sample size used for analysis when the data are split by sex.

**Table 4:** Results of generalized linear model selection for outer layer blubber cortisol concentrations (*n* = 20) showing the four best‐fitting models with the lowest second‐order Akaike information criterion (AICc) values

Model	Variables	*df*	AICc	ΔAICc	Weight
1	Sex + age class	4	209.4	0.0	0.29
2	Sex + mass/length^2^	4	210.9	1.5	0.14
3	Age class	3	211.9	2.5	0.08
4	Age class + sex + COD	5	212.2	2.8	0.07

Models 1 and 2 are of equivalent fit to the data. COD, cause of death.

Cause of death was not retained in the final model likely because it is tightly linked to the condition of the individuals as the acute cases were generally in better condition, and showed a smaller range in mass/length^2^ than the chronic cases with mean mass/length^2^ values of 0.0022 ± 0.0035 and 0.0018 ± 0.00050 for each group, respectively. The body condition of the animals therefore explained more of the variation in the data than the COD.

## 4 Discussion

### 4.1 Mass/length^2^ as a condition index

Mass/length^2^ was determined to be the most appropriate morphometric index for valid inferences about the condition of harbor porpoises. It showed good fit to the data, was consistent with the hypothesized relationships between both COD and age class, and was more independent of body size than mass/length alone. Correlations with body size are a problem because one main objective of condition indices is often to be able to compare the condition of animals of different sizes. The scaling of mass with length therefore needs to be considered when using indices that attempt to summarize the variation in mass relative to length, and attribute this variation to changes in condition.

There was little difference between the effect size of COD between the different condition indices, showing that it explained similar proportions of the variance in each model. Acute cases were in better body condition as these animals tended to have suffered a sudden critical event which led to their death, in comparison with the chronic cases where a general debilitation and decline in health occurred in individuals suffering from infectious disease or starvation. There was more variation explained by age class than COD in the mass/length^2^ model. As expected, based on the ground‐truthing assumptions, adults were in significantly better condition than both juveniles and calves. Juveniles were hypothesized to be in the poorest condition overall, and the fact that they were of intermediate condition here may be a result of how the individuals were assigned an age class following the work by Ólafsdóttir et al. (2002). Some of the smallest individuals may have only just been weaned, while others were almost at sexual maturity, resulting in a large range in the sizes of individuals in this age group.

Calves were shown to be in the poorest condition, but this would not necessarily imply that the calves are in poorer health. Rather, it more likely demonstrates that caution is required when comparing the condition of calves to the condition of adults using the same mass/length^2^ index as even though it is more independent of body size than mass/length in this species, it does not take into account rapid growth processes or the different proportion of mass associated with energy reserves or insulation for these two age classes of significantly different body sizes. This is likely also the case, although to a lesser extent, with smaller juveniles as energy allocation in porpoises has been shown to shift from an emphasis on developing an insulative blubber layer in young animals to preparing the body for annual reproduction at sexual maturity (McLellan et al., 2002). While the scaled mass index appeared to capture the changes in condition between different age classes in small mammals (Peig & Green, 2009), it was not a good fit to these harbor porpoise data. The mass/length^2^ index may be better suited to cetaceans than small mammals previously investigated as they are large with a relatively simple body shape, and their energy reserves in the blubber and muscle constitute a much larger proportion of their entire body mass, between 50% and 60% (McLellan et al., 2002), compared to energy reserves in terrestrial species.

The mass/length^2^ index was a better fit to the data than the ventral blubber thickness, and the two indices of blubber thickness in relation to girth and length. Blubber thickness was also found to be a poor index of condition in harbor porpoises in the Bay of Fundy (Read, 1990). This is likely because condition in these animals is not solely a function of blubber reserves, but also of muscle mass as starving animals begin to catabolize muscle as well (Stegall, McLellan, Dillaman, Read, & Pabst, 1999). The reasons for this are likely twofold. First, the blubber must maintain a certain thickness to fulfill its other roles in thermoregulation, streamlining, and buoyancy (Gómez‐Campos, Borrell, & Aguilar, 2011), so the extent to which it can be reduced in thickness during nutritional stress is limited. Second, starving animals may begin to catabolize muscle to access vital proteins (Stegall et al., 1999), and for this reason, starved porpoises have likely depleted significant amounts of lean tissue. By using a body condition index that includes mass, other structural body components, like skeletal muscle, are taken into account as these will also be affected by food limitation and are therefore more representative of overall condition.

There were no differences in condition between males during the breeding season and over the rest of the year using the mass/length^2^ index. In theory, males could be in poorer condition during the breeding season as a result of the increased energetic demands associated with reproduction. For example, mature individuals are thought to seasonally allocate up to 4% of their total body mass to testicular growth (Neimanis, Read, Foster, & Gaskin, 2000). However, a decrease in condition over the breeding season for sexually mature males has not been reported as it has for females. It is expected that the mass/length^2^ index is the most informative body condition index for females as well as males, and differences in the reproductive state of females between resting, pregnant, and lactating would be detected (Lockyer, 1995b), thus distinguishing between breeding and nonbreeding animals.

### 4.2 Blubber cortisol concentrations across sampling sites

Based on the extraction efficiency and variability, an optimum sample mass of between 0.15 and 0.2 g of tissue was identified for cortisol extraction using these methods. Larger samples have poorer extraction efficiency as both the homogenization of the tissue becomes less efficient with larger pieces and the mass/solvent ratio is no longer optimal for efficient cortisol extraction. Smaller sample masses will produce inflated cortisol concentration estimations as a result of the error associated with small measurements, and then the multiplication effect as sample mass is used to estimate the concentration in ng/g of tissue. Although biopsy samples are not routinely collected from harbor porpoises, they are from larger cetacean species, and if the findings of this study are applicable across a range of species, then biopsy samples obtained from live animals should be within this mass range for reliable cortisol quantification using this extraction method.

Blubber cortisol concentrations measured in harbor porpoises here, between 3.65 and 759.51 ng/g, were higher than those previously reported in common dolphins (*Delphinus delphis*; Kellar et al., 2015) and belugas (*Delphinapterus leucas*; Trana et al., 2016) with reported average ranges of between 3.99–24.3 and 0.26–1.76 ng/g for each of these species, respectively. The order of magnitude differences between the maximum concentrations measured in each of these three studies could be a result of species‐specific differences in hormone concentrations and metabolism, as well as method alterations in the extraction and quantification processes between studies.

Here, no significant variation in blubber cortisol concentrations was seen across the three different sampling locations. Dorsal sampling through the collection of remote biopsies could therefore provide information on typical blubber cortisol concentrations across this area of the body. These results are consistent with the notion that while harbor porpoises and other small odontocetes may selectively mobilize lipids unevenly across different areas (Koopman et al., 2002; Tornero, Borrell, Forcada, Pubill, & Aguilar, 2004), blubber composition remains relatively consistent across the body (Koopman et al., 2002).

### 4.3 Cortisol stratification through blubber depth

Evidence of stratification in cortisol concentration was seen through the blubber depth with highest concentrations measured in the middle and inner layers. Overall, the highest concentrations were measured in the inner layer which was also found in belugas (Trana et al., 2015). This is perhaps because the inner layers are more highly vascularized in shallow diving odontocetes compared to the superficial blubber layers closer to the skin (McClelland et al., 2012). Inner layers are also closer to larger blood vessels that supply the underlying muscle tissue. Together, these likely result in a higher concentration of cortisol from the circulatory system in this part of the blubber.

This stratification in cortisol concentration is unsurprising given that cetacean blubber is stratified into three layers that can be differentiated visually, histologically, and biochemically in many cetacean species (Olsen & Grahl‐Nielsen, 2003; Smith & Worthy, 2006). In harbor porpoises specifically, the inner and middle blubber layers are more dynamic and are likely used for lipid deposition and mobilization at a much faster rate than the outermost layer next to the skin (Koopman, Iverson, & Gaskin, 1996). Given the differences in the rate of turnover of the tissue, the cortisol concentrations measured in the full‐depth blubber samples represent the integration of the hormone through the tissue over a longer time period than either the middle or inner layers alone. There were no significant differences in the concentrations measured in full‐depth blubber samples compared to the outer layer. Thus, these superficial samples could be representative of the longer‐term presence of cortisol in the blubber that is not subject to strong fluctuations as a result of short‐term changes in lipid mobilization or deposition, or changes in the blood flow through the tissue to the skin during thermoregulation (Barbieri et al., 2010; Noren, Williams, Berry, & Butler, 2009). Cortisol concentrations in the outer blubber layer available through remote dart biopsy could thus be used as a potential indicator of longer‐term physiological changes.

### 4.4 Cortisol concentrations as a potential marker of condition

Covariate analyses revealed that, even with a small sample size of just 20 individuals and high levels of individual variation, cortisol concentrations in dorsal, outer‐layer samples were weakly negatively correlated with body condition, higher concentrations were measured in juveniles than adults, and in females than in males. In terrestrial mammals (Castellini & Rea, 1992), humans (Bergendahl, Vance, Iranmanesh, Thorner, & Veldhuis, 1996), and pinnipeds (Champagne, Crocker, Fowler, & Houser, 2012; Champagne, Houser, & Crocker, 2006; Guinet et al., 2004), extended periods of food restriction are associated with an increase in the circulating concentrations of cortisol. During these periods of reduced food intake or fasting, cortisol increases lipolysis to provide energy (Bergendahl et al., 1996) and is involved in the maintenance of circulating glucose concentrations through increased gluconeogenesis (Exton et al., 1972). It is likely that the same principles apply to cetaceans, and as such, increased cortisol concentrations in the blubber of harbor porpoises in poorer condition could be a result of increased mobilization of fat reserves in these individuals. Similarly, higher concentrations measured in the blubber of juveniles could be as a result of these individuals mobilizing energy reserves to maximize growth to achieve size at sexual maturity (McLellan et al., 2002) instead depositing lipid stores. Juveniles are likely also under both more nutritional and physiological stress than adults associated with foraging independently for the first time and being immunologically challenged by new pathogens, again, leading to the mobilization rather than the deposition of fat stores. Blubber cortisol concentrations should therefore be interpreted with the multi‐functional roles of cortisol in the regulation of the distribution of fat deposits, adipogenesis, and adipose metabolic and endocrine function in mind (Lee, Pramyothin, Karastergiou, & Fried, 2014), rather than as a passive hormonal store with no metabolic effect on the tissue itself.

Steroids are known to diffuse passively from the blood into adipose tissue (Deslypere, Verdonck, & Vermeulen, 1985), but the turnover of these hormones in human adipose tissue is slow (Hughes, Reynolds, Andrew, Critchley, & Walker, 2010). The turnover of cortisol in the blubber of marine mammals is unknown. As the blubber of shallow diving cetaceans has been shown to be more highly vascularized than the adipose tissue typical of terrestrial species (McClelland et al., 2012), a greater perfusion of the tissue could result in the high concentrations of cortisol measured here, but it is still unknown to what extent steroid hormones are in dynamic equilibrium between the blubber and the circulatory system. In addition, intraadipose cortisol is derived not only from the systemic circulation but also from the local conversion of its inert precursor, cortisone, into cortisol by the enzyme 11β‐hydroxysteroid dehydrogenase type 1 (Stimson et al., 2009). While it is unknown how much of the cortisol pool in cetacean blubber may be derived from local production, the cortisol concentrations measured in the blubber here may reflect a combination of both circulating concentrations of this hormone through passive diffusion from the blood, and the local production of the hormone in the tissue itself.

These results highlight the need to investigate the effect of the condition and physiological state of individuals before concluding that cortisol concentrations measured in the blubber are the result of heightened psychological stress alone. If these results persist across other cetacean species, the analysis of cortisol concentrations in remotely obtained blubber biopsies, the metabolic site of action of this hormone, has the potential to be a valuable tool for studying physiology and body condition particularly in free‐ranging cetaceans. More generally, given the importance of cortisol in the regulation of lipolysis and gluconeogenesis, concentrations in the adipose tissue of other mammalian species for which morphometric measurements cannot be obtained could also be of use as an informative condition marker. In turn, the ability to measure the condition of free‐ranging individuals has important implications for studying the population‐level effects of changing environments and exposure to increasing anthropogenic stressors.

## Conflict of Interest

None declared.
