# Supplementary material for: Evaluating morphometric and metabolic markers of body condition in a small cetacean, the harbor porpoise (Phocoena phocoena)
Source: Ecol Evol. 2017 Apr 9;7(10):3494–506. doi: 10.1002/ece3.2891 (PMC5433969; doi:10.1002/ece3.2891)
Supplement: Supplementary file 1 [file ECE3-7-3494-s001.pdf]

SUPPORTING INFORMATION

Evaluating morphometric and metabolic markers of body condition in a small cetacean, the harbour porpoise (*Phocoena phocoena*)

Kershaw, J. L.[1] Sherrill, M[1]. Davison, N[2]. Brownlow, A[2]. Hall, A. J.[1]

[1] Sea Mammal Research Unit, Scottish Oceans Institute, University of St Andrews,

St Andrews, Fife, KY16 8LB, UK.

[2] Scottish Marine Animal Stranding Scheme, SAC Veterinary Services Drummondhill, Stratherrick Road, Inverness, IV2 4JZ, UK

* Corresponding author: jk49@st-andrews.ac.uk

Supplementary Methods

ELISA Assay and Steriod Extraction Verifications

Quality assurance and quality control tests were performed to validate the use of this cortisol ELISA (DRG International Inc., Marburg, Germany : Cortisol ELISA EIA-1887) with harbour porpoise blubber extracts. Limitations and sources of error in the cortisol extraction method were also assessed so as to better interpret the results and assess the potential use of this method for remotely obtained biopsy samples.

Parallelism Assays: Three subsample extracts, from the outer, middle and inner layers were pooled and serially diluted four times from the undiluted sample to a 1/16 dilution. The resulting curve of the detection metric (optical density of the sample read at 450nm) as a function of the dilution state (1, ½, ¼, $^{1}/_{16}$) was then compared to the standard curve. Parallelism of these regression lines supports the assumption that the antigen binding characteristics allow the reliable determination of hormone levels in the diluted blubber extracts (Andreasson et al. 2015).

In order to assess parallelism, two linear regression models for dilution state against optical density, one with and one without an interaction with sample type (ELISA standard or blubber

extract), were compared (ANCOVA function in statistical package R, version 3.1.2, R Core Development Team, 2014). If the regression lines for each model do not have significantly different slopes, then these data indicate that the standard curve and the extract dilution curve are parallel, and the ELISA kit is reliably measuring cortisol in the tissue samples.

Matrix Effect Tests: PBS was confirmed to be a compatible sample diluent with this cortisol ELISA kit in previous work with blubber extracts (Kershaw and Hall 2016).

Extraction Efficiency: The extraction efficiency of the steroid extraction method was assessed across tissue samples of different masses between 0.1 and 0.3g, the typical range of sizes of biopsy samples obtained from free-ranging cetaceans. One full depth blubber sample was divided into masses of 0.05g, 0.1g, 0.15g, 0.2g, 0.25g, and 0.3g (all ±0.025g), each one in triplicate, such that one sample of each mass was unspiked while the other two were spiked with 100ng of cortisol. Cortisol was then extracted and measured as discussed above, and the average percent recovery calculated for each sample.

Minimum Sample Mass: To determine the minimum sample mass required to obtain robust, replicable measurements of cortisol concentrations in the blubber samples, multiple extractions from the same piece of tissue of differing masses were assayed. Again, full depth, duplicate subsamples of different masses between 0.05-0.3g at 0.05g intervals were taken from the same blubber sample. Cortisol was extracted and measured in these samples in tandem, and the mean cortisol concentration calculated for each one.

Supplementary Results

ELISA Assay and Steriod Extraction Verifications

Parallelism Assessment: The comparison between the two linear regression models, one with and one without an interaction with sample type showed that removing the interaction did not significantly affect the fit of the model (ANCOVA; $F = 10.78$, $p=0.103$). Therefore, we can conclude that the effect on optical density with increasing sample dilutions is the same for the standard samples and the blubber extracts, and that the regression lines are parallel (Fig. 1A). This ELISA is therefore suitable for the quantification of harbour porpoise cortisol.

Extraction Efficiency and Minimum Sample Mass: The extraction efficiencies ranged from an average of 88.4% for the 0.05g samples, down to an average of 75.7% for the 0.3g samples. However, the relationship between the extraction efficiency and sample mass was not linear (Fig. 1B). For this reason, a 4 parameter log-logistic model was fit to these data to model the effect of tissue mass on extraction efficiency (Fig. 1B). This model was used to calculate the expected extraction efficiencies for all the blubber subsamples processed in this study. These extraction efficiencies were used to correct the measured cortisol concentrations in each sample to give a final cortisol concentration used for statistical analysis. In terms of minimum sample size, as the sample mass increased, both the cortisol concentration measured and the variation between the duplicate subsamples decreased (Fig. 1C). Only the 0.15g, 0.2g and 0.25g subsamples overlapped in their measured cortisol concentrations (Fig. 1C). Thus, based on the decrease in extraction efficiency (Fig. 1B) and increase in measurement variability (Fig. 1C), it is recommended for maximum extraction efficiency and replicability, that blubber samples for extraction should be between 0.15 and 0.2g.

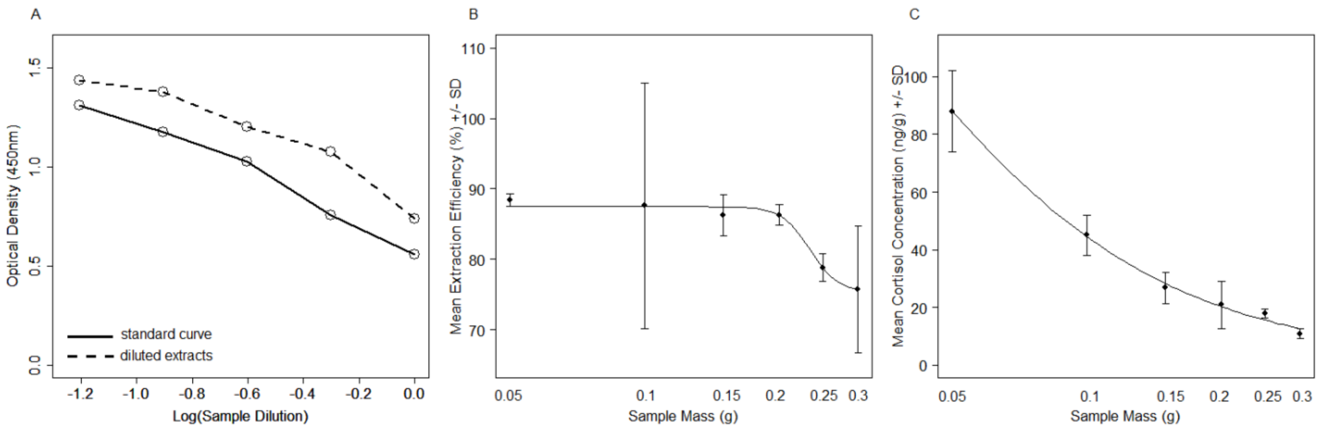

Supplementary Fig. 1 Quality assessment and control for cortisol quantification and extraction. **A.** Linearity assessment of the cortisol ELISA with harbour porpoise blubber tissue extracts. Serial dilutions of three pooled extracts show parallelism with the standard curve of the ELISA (ANCOVA $F = 10.783$, $p = 0.103$) **B.** Extraction efficiency from the same piece of tissue decreases with increasing blubber sample mass in a non-linear fashion. The extraction efficiency decreases with samples over 0.2g. **C.** Mean cortisol concentration in duplicate samples from the same piece of tissue decreases with increasing sample mass in a non-linear fashion. Measured concentrations overlap in sample masses between 0.15g and 0.25g.
